# Unraveling clinical and biological predictors of successful treatment free remission in Chronic Myeloid Leukemia and its impact on the healthcare system in a low- and middle-income country

**DOI:** 10.12669/pjms.42.6.15888

**Published:** 2026-06

**Authors:** Kaleem Ahmed, Sahrish Khan, Zafar Iqbal, Muhammad Arshad

**Affiliations:** 1 Kaleem Ahmed (PhD Researcher), Department of Biological Sciences, International Islamic University, Islamabad, Pakistan; 2 Sahrish Khan (PhD Researcher), Center for Applied Molecular Biology (CAMB), University of the Punjab, Lahore, Pakistan; 3 Prof. Dr. Zafar Iqbal (PhD), Center for Applied Molecular Biology (CAMB), University of the Punjab, Lahore, Pakistan; 4 Dr. Muhammad Arshad, Associate Professor, Department of Biological Sciences, International Islamic University, Islamabad, Pakistan

**Keywords:** Chronic Myeloid Leukemia, Deep molecular response, Tyrosine Kinase Inhibitors, Treatment Free Remission

## Abstract

**Objectives:**

Tyrosine Kinase Inhibitors (TKIs) have revolutionized the management of Chronic Myeloid Leukemia (CML), making treatment free remission (TFR) possible in eligible patients. However, TFR data in Pakistan remains scarce. This study aimed to evaluate relapse-free survival (RFS) outcomes and identify clinical predictors of successful TFR in CML patients.

**Methodology:**

Adult CML (chronic phase) patients > 18 years, eligible for TFR were included. It was an observational (cohort based study) and was conducted between 2022 and 2025 at Armed Forces Bone Marrow Transplant Center (AFBMTC), Rawalpindi. Minimum required duration of TKI was > 3 years and duration of DMR was > 2 years. Data was collected from bone marrow transplant center.

**Results:**

Thirty patients (16 males and 14 females) attempting TFR were analyzed. Median age at diagnosis was 34.5 years (range 19 – 60) and the median age at discontinuation was 38.5 years (range 24-63). Most patients had an intermediate SOKAL risk score (56.7%, n=17), while 30% (n=9) were low risk and 13.3% (n=4) had high risk. Imatinib was the most frequently used first-line TKI (83.3%, n=25), followed by nilotinib (16.7%, n=5). The median treatment duration of TKI was 40 months (range 36-60), and the median duration of sustained deep molecular response before attempting TFR was 30.5 months (range 24-49). RFS was 80% at six months and 66.7% at 12 months. Depth and duration of DMR and SOKAL scores positively defined the success of TFR with depth of DMR achieving significance levels. Relapsed patients (9 out of 10) had high or intermediate SOKAL risk scores.

**Conclusion:**

Despite limited data available for Pakistan, TFR remains a viable and safe option in carefully selected patients.

## INTRODUCTION

Chronic myeloid leukaemia (CML) is a major blood malignancy characterized by the expression of breakpoint cluster region/Abelson murine leukaemia viral oncogene 1 (BCR-ABL1).^1^ The prevalence of chronic myeloid leukemia (CML) is increasing, with estimates suggesting around five million people affected worldwide.^2^,^3^ Treatment for CML focuses on four primary goals:

- Enhancing overall survival;
- Achieving a sustained deep molecular response that could lead to treatment-free remission;
- Minimizing both short and long-term side effects; and
- Delivering value in treatment options. Currently, there are six approved BCR::ABL1 tyrosine kinase inhibitors (TKIs), five of which are used in frontline treatment (imatinib, dasatinib, bosutinib, nilotinib, and asciminib), while all six are applicable in later-line therapies, including ponatinib.^4^

Innovative third-generation TKIs targeting the ABL1 kinase domain, such as olverembatinib (ELVN-001) and TGRX-678 (TERN-701) focused on the myristoyl pocket, are also being developed. If not treated, CML can advance from a chronic phase (CP) to an accelerated phase (AP), and eventually to a blast phase (BP). The FDA’s approval of imatinib mesylate in 2001 marked a significant shift in how CML is managed, resulting in better survival rates in CML patients. TKI therapy has the potential to reduce CML to such low levels that it becomes undetectable through cytogenetic or hematologic tests, detectable only by molecular testing like quantitative polymerase chain reaction (qRT-PCR).^5^,^6^

Early achievement of minimal residual disease during TKI treatment signals a positive outlook for patients. The introduction of BCR-ABL tyrosine kinase inhibitors (TKIs) has fundamentally changed how we manage chronic myeloid leukemia (CML). As a result of the significant survival improvements linked to TKI therapy, we have witnessed a growing prevalence of CML, with forecasts indicating this trend to continue in the years ahead. Notably, patients with CML who respond well to TKI treatment are now enjoying life expectancies that approach those of the general population, especially among individuals as young as 55.^2^,^3^ Those diagnosed with chronic phase CML can now lead better quality lives as compared to those diagnosed in accelerated or blast phases. However, the long-term use of TKIs can come with side effects, high costs, and psychological challenges that may affect patients’ well-being. Consequently, the focus of treatment has shifted toward achieving treatment-free remission (TFR), which has emerged as a pivotal goal in managing chronic phase CML.^7^ Hematologists worldwide are increasingly considering TFR for patients they assess as suitable candidates. Research from developing countries reveals that around 40-50% of patients experience relapses after stopping TKI treatment. This is particularly concerning given that most CML patients reside in low- and middle-income countries, where access to TKIs and molecular testing is often limited, leading to adherence challenges.^8^

One of the earliest pivotal trials examining treatment-free remission (TFR) in chronic myeloid leukemia (CML) was the STIM trial.^7^ Following this, numerous other studies have evaluated TFR outcomes. Considering the increasing availability of such research, the European Leukemia Net (ELN) and the National Comprehensive Cancer Network (NCCN) have established guidelines to aid in selecting appropriate candidates for TFR.^9^-^12^ However, in resource-limited and underserved regions, these guidelines are not always fully applicable, leading to the need for individualized criteria for TFR implementation.^11^,^12^ The primary objective of our study was to identify clinical predictors associated with successful TFR. Our TFR criteria adhered to the recommendations outlined by the NCCN, ELN and TFR studies across the world. A prospective database was maintained for patients undergoing TFR, and outcome analysis was conducted on this data for individuals who initiated TFR up until December 2025. Ethical approval for the study was obtained from both the institutional ethics committee at the University and partnering Institute.

Certain patients respond early to treatments while others fail to do so, similarly few patients remain in TFR after stopping therapy while others relapse. In order to make the right treatment decisions and select the right patients for attempting TFR, it is critical to understand the predictors of sustained TFR and long term treatment response.

## METHODOLOGY

Our study was observational (cohort based study in which CML patients were followed over time to assess TFR outcomes) and data was collected from Armed Forces Bone Marrow Transplant Center (AFBMTC), Rawalpindi.

### Ethical approval:

The study was conducted between 2022 and 2025. Ethical approval was taken from both the University and participating center (Ref: IRB-009/AFBMTC/Approval/2023; dated September 20, 2023).

Following inclusion criteria was defined to initiate TFR. (a) CML in Chronic Phase, (b) Age >18 Years (c) any of the TKI as 1^st^ line therapy, (d) minimum TKI duration of therapy three years, (e) DMR for two years (Table-I). Regular monitoring with quantitative polymerase chain reaction was ensured by treating Physicians. A loss of MMR (BCR: ABL1 (IS) > 0.1%) was defined as the criterion for treatment re-initiation. Patients progressing to accelerated or blast crisis were excluded. We collected important clinical variables including age, gender, SOKAL Score, treatment duration, treatment response, depth and duration of DMR to identify predictors of TFR. Time to the loss of MMR and response to reinitiating of TKI therapy were obtained for relapse-free survival (RFS) analysis and safety evaluation, respectively. Our study was aligned with International guidelines on selection of right patients for TFR.

**Table-I:** Comparison of ELN, NCCN criteria and our study for initiation of TFR.

Parameter	NCCN guidelines	ELN guidelines	Our Study Criteria
1^st^ Chronic Phase	Yes	Yes	Yes
TKI	Any TKI, no specific dose	Any TKI, no specific dose	Any TKI, no specific dose
Minimum TKI duration	3 Years	5 Years	3 Years
DMR (MR ^4^ and MR^4.5^)	Minimum 2 Years	Minimum 2 Years	Minimum 2 Years

### Operational definitions:

In line with global studies and guidelines, our study defined major molecular response (MMR) as a 3 log reduction (MR3) in BCR::ABL1<0.1%, MR4 as a 4 log reduction or BCR::ABL1 ≤0.01%, and MR4.5 as a 4.5 log reduction or BCR::ABL1 ≤0.0032% in IS.

Successful treatment free remission was defined as eligible patients stopping TKIs and maintaining a DMR without relapsing till defined study timelines. The duration of TFR was calculated as the time between the date of discontinuation of TKI treatment and the date of resumption of TKI treatment or the date of last follow-up in the absence of recurrence. Molecular relapse or TFR failure was defined as loss of MMR (BCR::ABL1 > 0.1%).

Important clinical and biological predictors studied included; Age at diagnosis, age at TKI discontinuation, duration of TKI, depth and duration of DMR, Risk scores (ELTS and EUTOS), time to achieve molecular response, baseline WBCs, RBCs, Hemoglobin, Platelets and spleen size etc.

### Primary and secondary objectives:

Our primary objective was to determine the molecular relapse free survival (MRFS) which is time from discontinuation of therapy to relapse and to identify clinical predictors associated with successful TFR. Secondary objectives included observing patterns of relapse and prospects of re-initiating therapy after relapse.

### Statistical analysis:

The categorical variables like gender and Sokal score were summarized using the frequency and percentages. The quantitative variables were summarized as mean and standard deviation, or median with ranges, and quartiles.

## RESULTS

**Fig.1:**
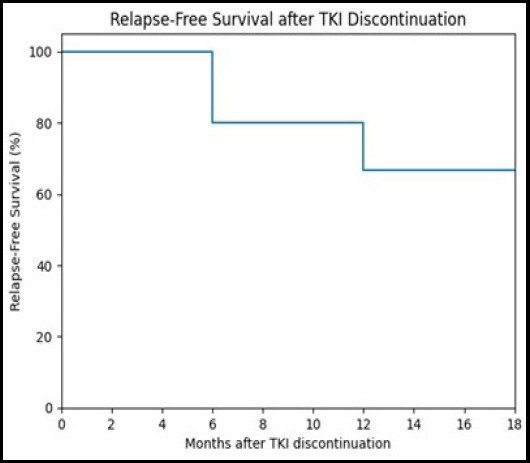
RFS outcomes of the patients attempting TKI Discontinuation.

Survival curves were estimated using the Kaplan-Meier method (Fig.1). The potential prognostic effect of covariates for RFS is estimated using univariate and multivariate Cox proportional hazard models and reported by applying the two-sided log-rank test, and P-values < 0.05 are considered statistically significant. The results showed significance levels attained for depth of deep molecular response.

Our cohort had 30 carefully selected patients who discontinued TKI therapy (Table-II). The table describes the baseline clinical and treatment characteristics of these 30 patients with chronic myeloid leukemia (CML). The cohort included 14 females and 16 males. The median age at CML diagnosis was 34.5 years (range, 19–60), and the median age at TKI discontinuation was 38.5 years (range, 24–63), representing prevalence at a younger age which is unique to our population. SOKAL score was calculated from patients’ clinical parameters. According to which, nine patients were classified as low risk, seventeen as intermediate risk, and four as high risk. This is in line with previous studies which in which TKI discontinuing patients had scores in low and intermediate risk categories.

**Table-II:** Baseline Characteristics and Demographics of CML Patients Discontinuing Treatment.

Parameter	Values n= 30
Female/Male	14/16
Age (y) at CML diagnosis, median (range)	34.5 (19-60)
Age at TKI stop, median (range)	38.5 (24-63)
***Sokal score, n (%)***	
Low	9
Intermediate	17
High	4
***First-line TKI, n (%)***	
Imatinib	25 (83.3)
Dasatinib	0
Nilotinib	5 (16.6)
***Second-line TKI, n (%)***	
None	12 (40)
Nilotinib	12 (40)
Dasatinib	6 (20)
Imatinib	0
***Last TKI before discontinuation, n (%)***	
Imatinib	10 (33.3)
Nilotinib	14 (46.6)
Dasatinib	6 (20)
Time (mo) to achieve initial MMR, median (IQR)	11.5 (7-15)
Total duration of TKI therapy (mo), median (IQR)	40 (36-60)
Duration of sustained DMR before TKI discontinuation (mo), median (IQR)	30.5 (24-49)
Molecular response at discontinuation of TKI, n (%)	100
Follow up after TKI discontinuation (mo), median (IQR)	18 (4-30)

Imatinib was the most commonly used first-line TKI (n=25), followed by nilotinib (n=5), while no patients received dasatinib as first-line therapy. Eighteen patients required second-line therapy, most frequently nilotinib (n=12) and dasatinib (n=6). At the time of TKI discontinuation, the last TKI used was nilotinib in 14 patients, imatinib in 10 patients, and dasatinib in six patients.

The median time to achieve initial major molecular response (MMR) was 11.5 months (IQR, 7–15). The median total duration of TKI therapy was 40 months (IQR, 36–60), and the median duration of sustained deep molecular response (DMR) prior to TKI discontinuation was 30.5 months (IQR, 24–49). These durations were in line with NCCN and ELN guidelines. At discontinuation, all patients had achieved DMR. The median follow-up after TKI discontinuation was 18 months (IQR, 4–30).

In our cohort, 10 out of 30 patients relapsed. The clinical characteristics and patterns of patients who experienced molecular relapse following tyrosine kinase inhibitor (TKI) discontinuation are summarized in Table-III. Out of ten patients, four were females and six were males. The median age at relapse was 38 years (range, 25–60). According to Sokal risk stratification, the majority of relapsed patients were classified as intermediate risk (n=7), followed by high risk (n=2), while only one patient was low risk. This is in line with most of the TFR studies, reporting higher probability of relapses in patients having intermediate to high risk Sokal score.^13^,^14^

**Table-III:** Characteristics of CML patients who relapsed after TKI discontinuation.

Parameter	Values
Number of patients relapsed, n (%)	10 (33.3)
Median age (y) at relapse (range)	38 (25-60)
Gender: Female/Male	4/6
***Sokal score***	
High	2
Intermediate	7
Low	1
Total duration (mo) of TKI therapy, median (range)	44 (36-48)
***Last TKI at discontinuation***	
Imatinib	5
Dasatinib	0
Nilotinib	5
Time (mo) to relapse, median (range)	5.3 (4-7)
***Time periods of relapse (n = 10)***	
0-6 mo	6
6-12 mo	4
Time (mo) to achieve MMR after re-initiation of TKI, median (range)	4 (2-6)

The median total duration of TKI therapy prior to discontinuation among relapsed patients was 44 months (range, 36–48). At the time of TKI cessation, the last TKI administered was imatinib in five patients and nilotinib in five patients; no relapses occurred in patients discontinuing dasatinib. Dasatinib has been linked with immunomodulatory effects, including expansion of the cytotoxic NK cells, which may have contributed to successful TFR, however, since the population size was medium in our case, so more data and insights need to be gained on this aspect before making any opinion on Dasatinib’s superiority versus other TKIs.

Relapses occurred predominantly within the first year after TKI discontinuation. Six patients relapsed within the first six months, and an additional four patients relapsed between six and 12 months. Following re-initiation of TKI therapy, all patients regained major molecular response (MMR) in a median time of four months, consistent with previously reported TFR experiences.^15^,^16^

Our study revealed that depth of deep molecular response was the most significant factor associated with successful treatment free remission. Patients having MR4.5 were less vulnerable to relapse as compared to those having MR4. Our findings are in line with many regional and global trials outlining similar outcomes.^17^-^19^ Other factors which had positive and meaningful implications towards probability of attaining TFR were long duration of TKI and low SOKAL score.

## DISCUSSION

In our study, 30 patients with Chronic Myeloid Leukemia attempting TFR were included. 20 of these patients had achieved successful TFR till last follow-up, whereas 10 relapsed. The most significant factor associated with successful treatment free remission was depth of molecular response. Those with MR^4^,^5^ had a better chance of staying in remission as compared to those with MR.^4^ Our study establishes the fact that Treatment Free Remission is possible in carefully selected patients. Certain clinical and biological predictors can help identify the right patients for TFR. This requires identifying the right patients based on certain clinical predictors and ensuring strict molecular monitoring. Low Sokal score, longer TKI duration, depth and duration of DMR are key factors reported to influence the success of TFR. In our study, depth and duration of DMR, Sokal score and TKI duration were important factors associated with successful TFR, however only depth of DMR was statistically significant.

Our findings are supported by a recent systematic review and meta-analysis.^19^ Studies done in regional and Pakistani patients suggest positive prospects for initiating TFR.^20^,^21^ Also, few studies have found a correlation with baseline WBCs and spleen size on treatment outcomes.^20^ Treatment free remission (TFR) is now an important goal in the management of CML. Multiple clinical trials of TKI discontinuation in CML have been published^22^-^26^, still the real-world data are scanty, particularly from outside of Western countries. It is important to study TFR in different patient populations to know the dynamics and outcome of TKI discontinuation. Few of the important predictors from landmark trials are mentioned in Table-V. The results of the current study demonstrate that TKI discontinuation is feasible and safe in routine clinical practice and TFR can be achieved in two-third of carefully selected patients.

**Table-IV:** Univariate analysis of risk factors for molecular relapse after TKI discontinuation.

Variable	Category	Odds Ratio (95% CI)	P value
Age at discontinuation (median 45 yrs)	> 45 years Vs ≤ 45 years	1.28 (0.53–3.08)	0.58
Gender	Male VsFemale	0.89 (0.36–2.20)	0.80
Splenomegaly	Moderate / Massive Vs None/Mild	0.69 (0.27–1.76)	0.44
Sokal score	Intermediate / High Vs Low	0.73 (0.29–1.83)	0.50
EUTOS score	High vs Low	0.66 (0.21–2.06)	0.47
Time to initial MMR	> 12 months Vs <12 months	0.52 (0.21–1.30)	0.16
Depth of molecular response	MR4 Vs MR4.5	0.31 (0.12–0.78)	0.01
Total TKI duration	< 48 months Vs >48 months	0.55 (0.22–1.37)	0.19
Duration of sustained DMR	< 36 months Vs >36 months	0.81 (0.32–2.04)	0.66
3-month molecular response	Suboptimal Vs Optimal	0.49 (0.19–1.25)	0.13

**Table-V:** Important TKI Discontinuation Trials and Key Predictors of successful TFR.18,23-28

Study	TKI treatment (duration)	N	DMR criteria	Relapse criteria	RFS (12 months)	RFS (24 months)	Predictors of successful TFR
HAS	Imatinib or Nilotinib (3 years)	57	MR4 (2 years)	MMR	63.2%	56.8%	MR5 / CMR
KID	Imatinib (3 years)	90	MR5 (2 years)	MMR	62.2%	58.8%	Long TKI duration; dPCR negative; withdrawal syndrome
STIM1	Imatinib (3 years)	100	MR5 (2 years)	MR5	41%	38%	Male; long TKI duration; low Sokal score
TWISTER	Imatinib (3 years)	40	MR4.5 (2 years)	MMR	52%	45%	Long interferon use; short time to MR4.5
EURO-SKI	Any TKI (3 years)	728	MR4 (1 year)	MMR	53.5%	50.1%	Long TKI duration; low PB blasts count; e14a2 transcript
ENEST Freedom	Nilotinib (2 years)	190	MR4.5 (1 year)	MMR	51.6%	48.9%	Low Sokal score
LAST	Any TKI (3 years)	171	MR4 (2 years)	MMR	65.7%	60.8%	Deep MR; dPCR negative

A review of studies on TKI discontinuation^18^–^25^ highlights variations in patient selection criteria, monitoring practices, and post-TFR failure management. While most TFR studies report long-term MRFS rates between 40% and 60%, one study^25^ observed a notably higher MRFS of 79%. In this particular study, the median duration of TKI therapy before discontinuation was around 10 years, with 99% of patients achieving MR4.5 at the point of discontinuation. Numerous studies have also explored the influence of both patient-specific and disease-related factors on long-term TFR outcomes.^26^-^28^ As summarized in other studies, factors such as age, first-line therapy, treatment duration prior to TFR, the length of MR4 or MR4.5 before stopping TKI, Sokal score, and quantitative PCR values from droplet digital PCR before discontinuation have been identified as important predictors.^27^,^28^ These findings emphasize that patient selection criteria play a crucial role in determining long-term TFR success. This consideration is particularly significant in low- and middle-income countries (LMICs), where logistical challenges hinder rigorous monitoring processes.

As TFR in CML becomes increasingly integrated into routine clinical care, our regional study and experience offers valuable insights into patient selection, response monitoring, and long term outcomes. Such evidences coming from low and middle income countries contribute significantly towards the feasibility of TFR implementation in Asian patient populations, where continuous focusis on reducing the long-term treatment costs and healthcare burden. Successful TFR has the potential to significantly reduce overall financial and treatment burden.

Our study contributes data from a real world Pakistani patient population, which still is underrepresented in medical literature. Our study investigated predictors of both successful TFR and relapse, helping provide insights into both aspects.

### Limitations:

Since TFR is still a new concept in CML, so the small number of patients and short follow-up duration may have limited statistical power. Also, since the study was done in a low and middle income setting, so the cost constraints and delayed lab monitoring in few cases remained a challenge.

## CONCLUSION

TFR has become a new milestone in the management of CML Understanding the predictors of TFR can help identify the right patients for safely stopping the TKIs. Our study suggests that successful TFR can be achieved in two thirds of carefully selected patients. Patients who achieved a greater depth of molecular response had a better chance of successful TFR.

### Authors` Contribution:

**KA:** Data acquisition, drafting of manuscript, analysis and interpretation of data. He is responsible for accountability, data accuracy and integrity of work.

**SK:** Data acquisition, drafting of manuscript, critical review, analysis and interpretation of data.

**ZI:** Conceived and designed the study, supervised the work, provided technical and statistical support along with critical revision.

**MA:** Provided study supervision, critical review, guidance and support throughout the study.

All authors have read and approved the final version of manuscript.

## References

[1] Khoury JD, Solary E, Abla O, Akkari Y, Alaggio R, Apperley JF (2022). The 5th edition of the World Health Organization classification of haematolymphoidtumours: myeloid and histiocytic/dendritic neoplasms. Leukemia.

[2] Bower H, Bjorkholm M, Dickman PW, Hoglund M, Lambert PC, Andersson TM (2016). Life expectancy of patients with chronic myeloid leukemia approaches the life expectancy of the general population. J Clin Oncol.

[3] Jabbour E, Kantarjian H (2024). Chronic myeloid leukemia: 2025 update on diagnosis, therapy, and monitoring. Am J Hematol.

[4] Apperley JF, Milojkovic D, Cross NC, Hjorth-Hansen H, Hochhaus A, Kantarjian H (2025). European LeukemiaNet recommendations for the management of chronic myeloid leukemia. Chronic Myeloid Leukemia. Leukemia.

[5] Deininger M, O'Brien SG, Guilhot F, Goldman JM, Hochhaus A, Hughes TP (2009). International randomized study of interferon vs STI571 (IRIS) 8-year follow up: sustained survival and low risk for progression or events in patients with newly diagnosed chronic myeloid leukemia in chronic phase (CML-CP) treated with imatinib. Blood.

[6] Druker BJ, Guilhot F, O'Brien SG, Gathmann I, Kantarjian H, Gattermann N (2006). Five-year follow-up of patients receiving imatinib for chronic myeloid leukemia. N Engl J Med.

[7] Mahon FX, Réa D, Guilhot J, Guilhot F, Huguet F, Nicolini F (2010). Discontinuation of imatinib in patients with chronic myeloid leukaemia who have maintained complete molecular remission for at least 2 years: the prospective, multicentre Stop Imatinib (STIM) trial. Lancet Oncol.

[8] Giles FJ, Masszi T, Casares MTG, Hellmann A, Stentoft J, Conneally E (2019). Treatment-free remission (TFR) following frontline (1L) nilotinib (NIL) in patients (pts) with chronic myeloid leukemia in chronic phase (CML-CP): 192-week data from the ENEST freedom study. J Clin Oncol.

[9] Saussele S, Richter J, Guilhot J (2018). Discontinuation of tyrosine kinase inhibitor therapy in chronic myeloid leukaemia (EURO-SKI): a prespecified interim analysis of a prospective, multicentre, non-randomised, trial. Lancet Oncol.

[10] Shah NP, García-Gutiérrez V, Jiménez-Velasco A, Larson S, Saussele S, Rea D (2020). Dasatinib discontinuation in patients with chronic-phase chronic myeloid leukemia and stable deep molecular response: the DASFREE study. Leuk Lymphoma.

[11] Hochhaus A, Baccarani M, Silver RT, Schiffer C, Apperley JF, Cervantes F (2020). European LeukemiaNet 2020 recommendations for treating chronic myeloid leukemia. Leukemia.

[12] Deninger MW, Shah NP, Altman JK, Berman E, Bhatia R, Bhatnagar B (2020). Chronic myeloid leukemia, version 2.2021, NCCN clinical practice guidelines in oncology. J Natl Compr Cancer Netw.

[13] Wajid AA, Zeeshan M, Mehmood F, Sharif I, Umair M, Ali A (2018). Early molecular response with imatinib therapy in chronic myeloid leukemia and its association with baseline white blood cell count and spleen size. Pak Armed Forces Med J.

[14] Shahid S, Sabar MF, Iqbal Z, Saleem MZ, Khokhar MA, Ashiq S (2023). Molecular biology and drug therapies of chronic myeloid leukemia: a review of recent literature. Int J Pharm Integr Health Sci.

[15] Atallah E, Sweet K (2021). Treatment-free remission: the new goal in CML therapy. Curr Hematol Malig Rep.

[16] Rousselot P, Loiseau C, Delord M, Cayuela JM, Spentchian M (2020). Late molecular recurrences in patients with chronic myeloid leukemia experiencing treatment-free remission. Blood Adv.

[17] Dengler J, Tesch H, Jentsch-Ullrich K, Gerhardt A, Schulte C, Lipke J (2022). Treatment-free remission in real-world chronic myeloid leukemia patients: insights from german hematology practices. Acta Haematol.

[18] Han JJ (2023). Treatment-free remission after discontinuation of imatinib, dasatinib, and nilotinib in patients with chronic myeloid leukemia. Blood Res.

[19] Zheng Z, Tang H, Zhang X, Zheng L, Yin Z, Zhou J (2024). Treatment-free remission after discontinuation of tyrosine kinase inhi.bitors in patients with chronic myeloid leukemia in the chronic phase: A systematic review and meta-analysis. Discov Oncol.

[20] Khan U, Raza S, Mahmood A (2021). Molecular monitoring and relapse patterns after tyrosine kinase inhibitor discontinuation in Pakistani CML patients. Pak J Med Sci.

[21] Saeed N, Iqbal J, Hussain K (2022). Feasibility of treatment discontinuation in chronic myeloid leukemia in a resource-limited setting of Pakistan. Asian Pac J Cancer Prev.

[22] Hochhaus A, Masszi T, Giles FJ, Radich JP, Ross DM, Gómez Casares MT (2017). Treatment-free remission following frontline nilotinib in patients with chronic myeloid leukemia in chronic phase: results from the ENESTfreedom study. Leukemia.

[23] Okada M, Imagawa J, Tanaka H, Nakamae H, Hino M, Murai K (2018). Final 3-year results of the dasatinib discontinuation trial in patients with chronic myeloid leukemia who received dasatinib as a second-line treatment. Clin Lymphoma Myeloma Leuk.

[24] Atallah E, Schiffer CA, Radich JP, Weinfurt KP, Zhang MJ, Pinilla-Ibarz J (2021). Assessment of outcomes after stopping tyrosine kinase inhibitors among patients with chronic myeloid leukemia: a nonrandomized clinical trial. JAMA Oncol.

[25] Ross DM, Branford S, Seymour JF, Schwarer AP, Arthur C, Yeung DT (2013). Safety and efficacy of imatinib cessation for CML patients with stable undetectable minimal residual disease: results from the TWISTER study. Blood, J Am Soc Hematol.

[26] Lee SE, Choi SY, Song HY, Kim SH, Choi MY, Park JS (2016). Imatinib withdrawal syndrome and longer duration of imatinib have a close association with a lower molecular relapse after treatment discontinuation: the KID study. Haematologica.

[27] Mahon FX, Pfirrmann M, Dulucq S, Hochhaus A, Panayiotidis P, Almeida A (2024). European stop tyrosine kinase inhibitor trial (EURO-SKI) in chronic myeloid leukemia: final analysis and novel prognostic factors for treatment-free remission. J Clin Oncol.

[28] Saussele S, Richter J, Guilhot J (2018). Discontinuation of tyrosine kinase inhibitor therapy in chronic myeloid leukaemia (EURO-SKI): a prespecified interim analysis of a prospective, multicentre, non-randomised, trial. Lancet Oncol.

